# Supplementary material for: Deletion of a non-canonical regulatory sequence causes loss of Scn1a expression and epileptic phenotypes in mice
Source: Genome Med. 2021 Apr 26;13:69. doi: 10.1186/s13073-021-00884-0 (PMC8080386; doi:10.1186/s13073-021-00884-0)
Supplement: Supplementary file 1 — Additional file 1: Supplementary Figures. Figure S1. Tissue and brain regional differences in chromatin conformation. Figure S2. Western blots of NaV1.1 (250 kDa) and β-actin (45 kDa) proteins in P29–32 brain lysates, membrane and cytoplasmic fractions ran separately. Figure S3. Immunofluorescent analysis of sagittal sections of P28 mice. Figure S4. Seizure severity following Pentylenetetrazole (PTZ) administration in C57BL6/N mice. Figure S5. Differential gene expression with Scn1a 1b deletion in P32 hippocampus. [file 13073_2021_884_MOESM1_ESM.pptx]

## Slide 1
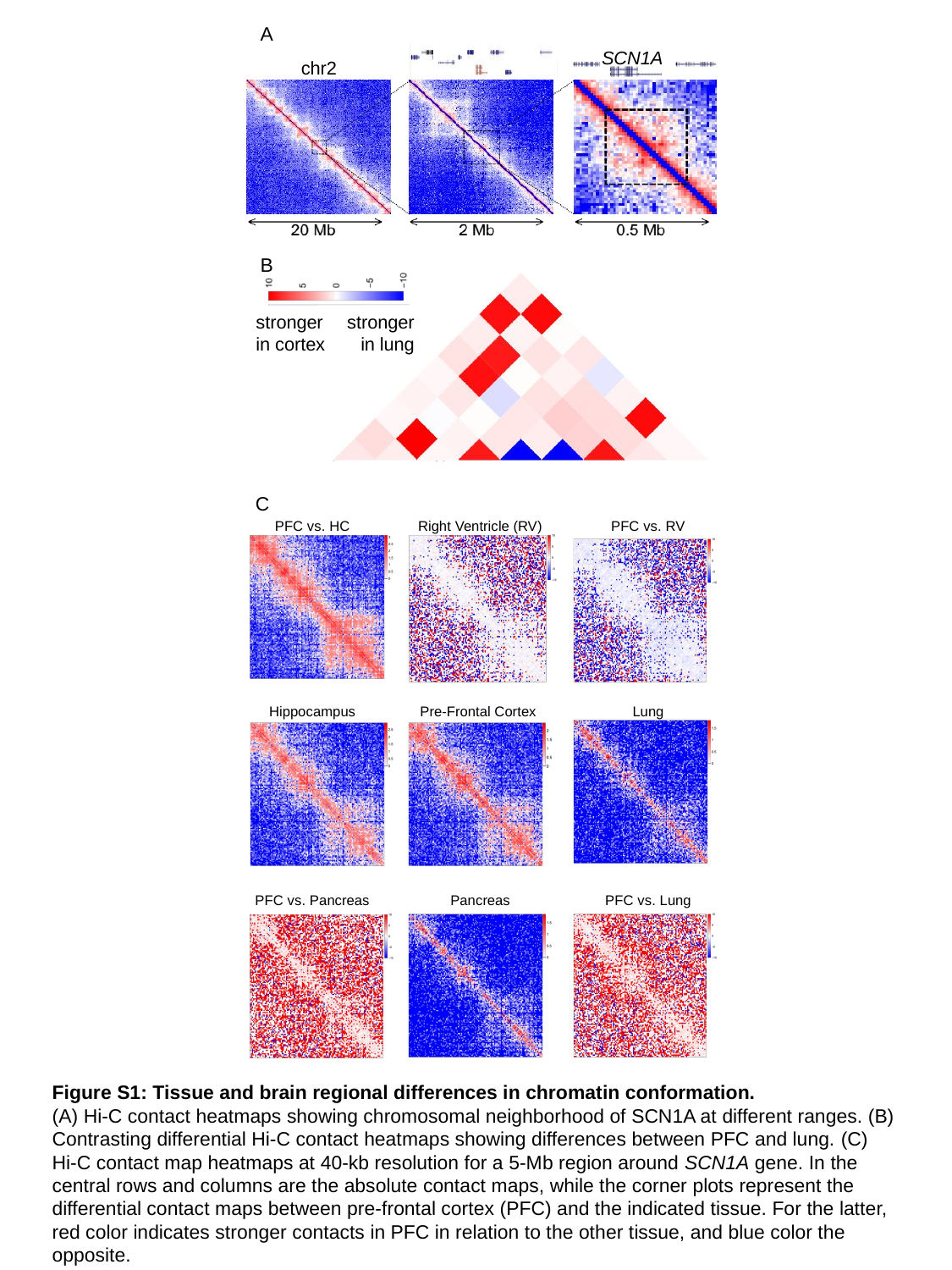

A
SCN1A
chr2
B
stronger
in lung
stronger
in cortex
C
| PFC vs. HC | Right Ventricle (RV) | PFC vs. RV |
| --- | --- | --- |
| Hippocampus | Pre-Frontal Cortex | Lung |
| PFC vs. Pancreas | Pancreas | PFC vs. Lung |
Figure S1: Tissue and brain regional differences in chromatin conformation.
(A) Hi-C contact heatmaps showing chromosomal neighborhood of SCN1A at different ranges. (B) Contrasting differential Hi-C contact heatmaps showing differences between PFC and lung. (C) Hi-C contact map heatmaps at 40-kb resolution for a 5-Mb region around SCN1A gene. In the central rows and columns are the absolute contact maps, while the corner plots represent the differential contact maps between pre-frontal cortex (PFC) and the indicated tissue. For the latter, red color indicates stronger contacts in PFC in relation to the other tissue, and blue color the opposite.

## Slide 2
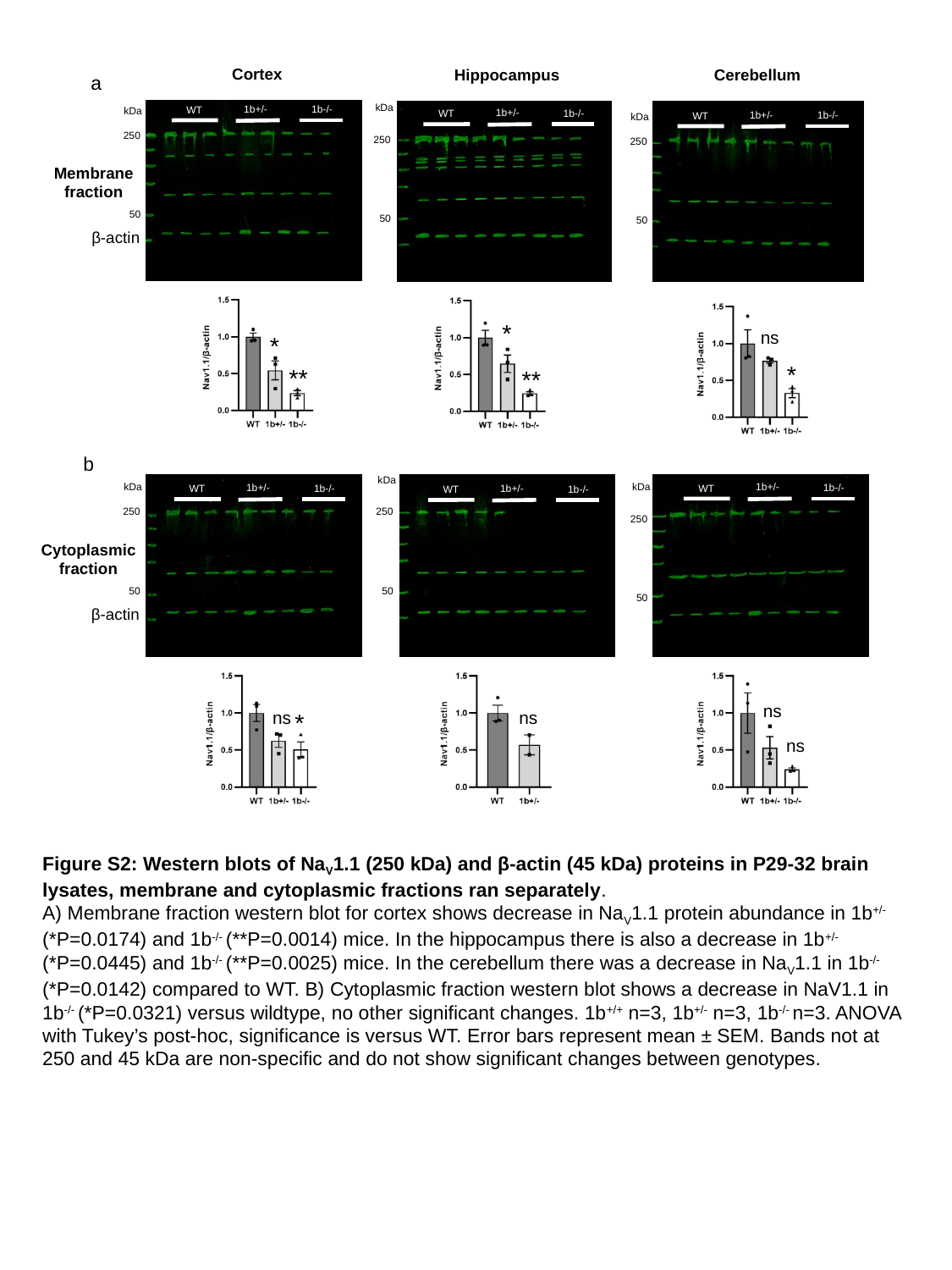

Cortex
Hippocampus
Cerebellum
a
kDa
1b+/-
1b-/-
WT
kDa
1b+/-
1b-/-
WT
1b+/-
1b-/-
WT
kDa
250
250
250
250
Membrane
fraction
50
50
B-actin
B-actin
50
50
50
β-actin
*
ns
*
*
**
**
b
kDa
kDa
kDa
1b+/-
1b+/-
1b-/-
WT
1b-/-
1b+/-
WT
1b-/-
WT
250
250
250
Cytoplasmic
fraction
50
50
50
β-actin
ns
ns
ns
*
ns
Figure S2: Western blots of NaV1.1 (250 kDa) and β-actin (45 kDa) proteins in P29-32 brain lysates, membrane and cytoplasmic fractions ran separately.
A) Membrane fraction western blot for cortex shows decrease in NaV1.1 protein abundance in 1b+/- (*P=0.0174) and 1b-/- (**P=0.0014) mice. In the hippocampus there is also a decrease in 1b+/- (*P=0.0445) and 1b-/- (**P=0.0025) mice. In the cerebellum there was a decrease in NaV1.1 in 1b-/- (*P=0.0142) compared to WT. B) Cytoplasmic fraction western blot shows a decrease in NaV1.1 in 1b-/- (*P=0.0321) versus wildtype, no other significant changes. 1b+/+ n=3, 1b+/- n=3, 1b-/- n=3. ANOVA with Tukey’s post-hoc, significance is versus WT. Error bars represent mean ± SEM. Bands not at 250 and 45 kDa are non-specific and do not show significant changes between genotypes.

## Slide 3
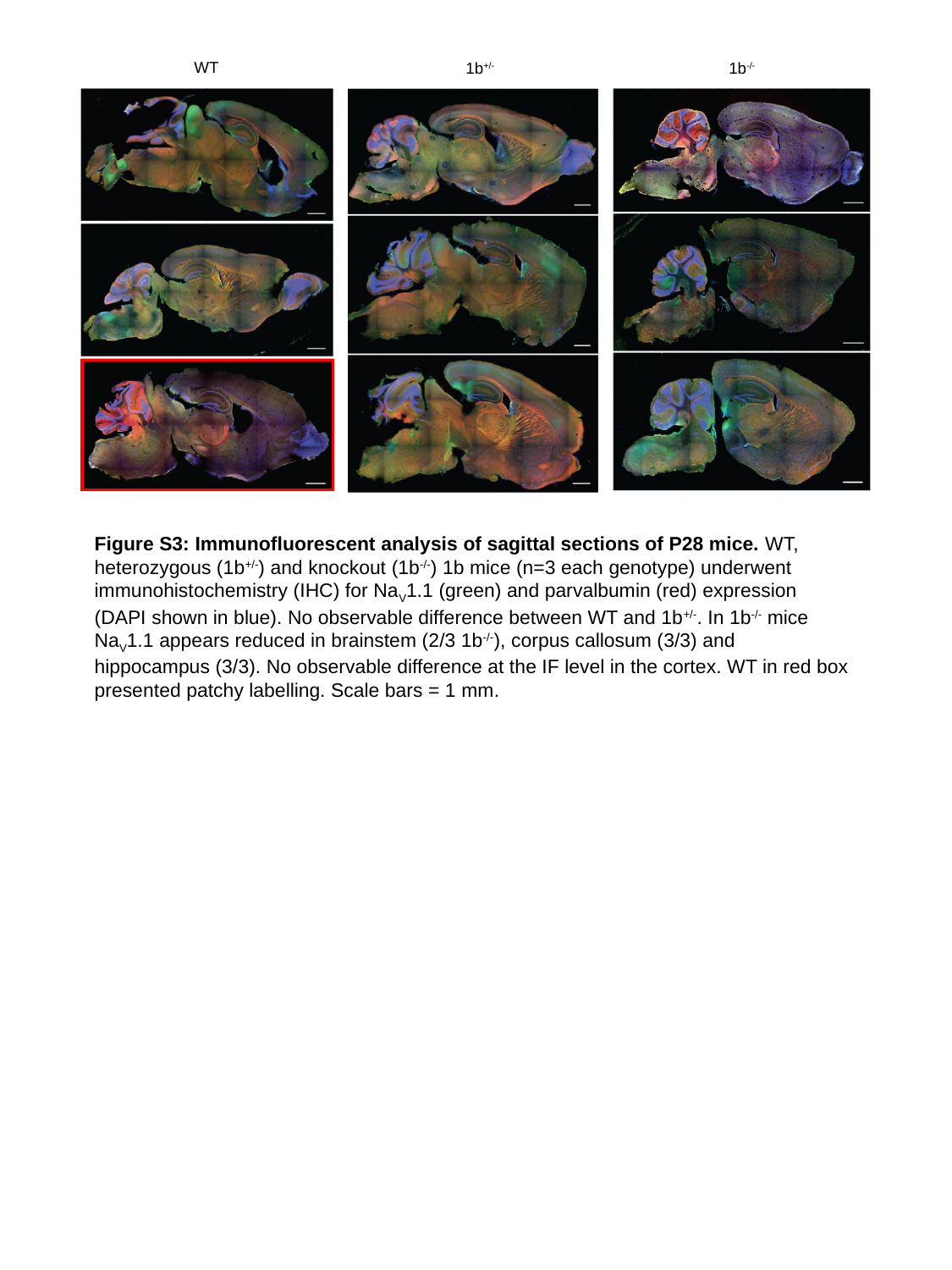

WT
1b+/-
1b-/-
Figure S3: Immunofluorescent analysis of sagittal sections of P28 mice. WT, heterozygous (1b+/-) and knockout (1b-/-) 1b mice (n=3 each genotype) underwent immunohistochemistry (IHC) for NaV1.1 (green) and parvalbumin (red) expression (DAPI shown in blue). No observable difference between WT and 1b+/-. In 1b-/- mice NaV1.1 appears reduced in brainstem (2/3 1b-/-), corpus callosum (3/3) and hippocampus (3/3). No observable difference at the IF level in the cortex. WT in red box presented patchy labelling. Scale bars = 1 mm.

## Slide 4
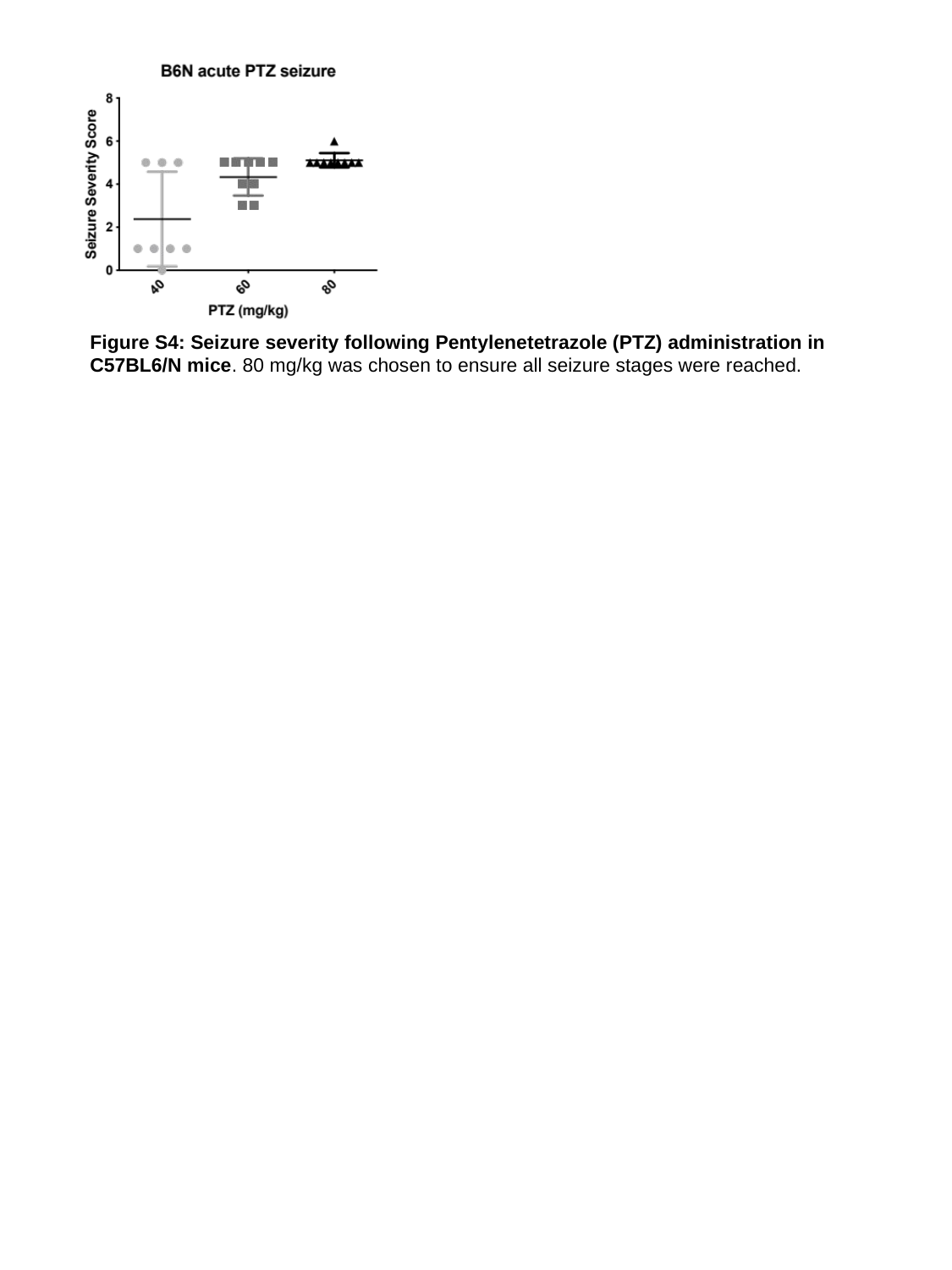

Figure S4: Seizure severity following Pentylenetetrazole (PTZ) administration in C57BL6/N mice. 80 mg/kg was chosen to ensure all seizure stages were reached.

## Slide 5
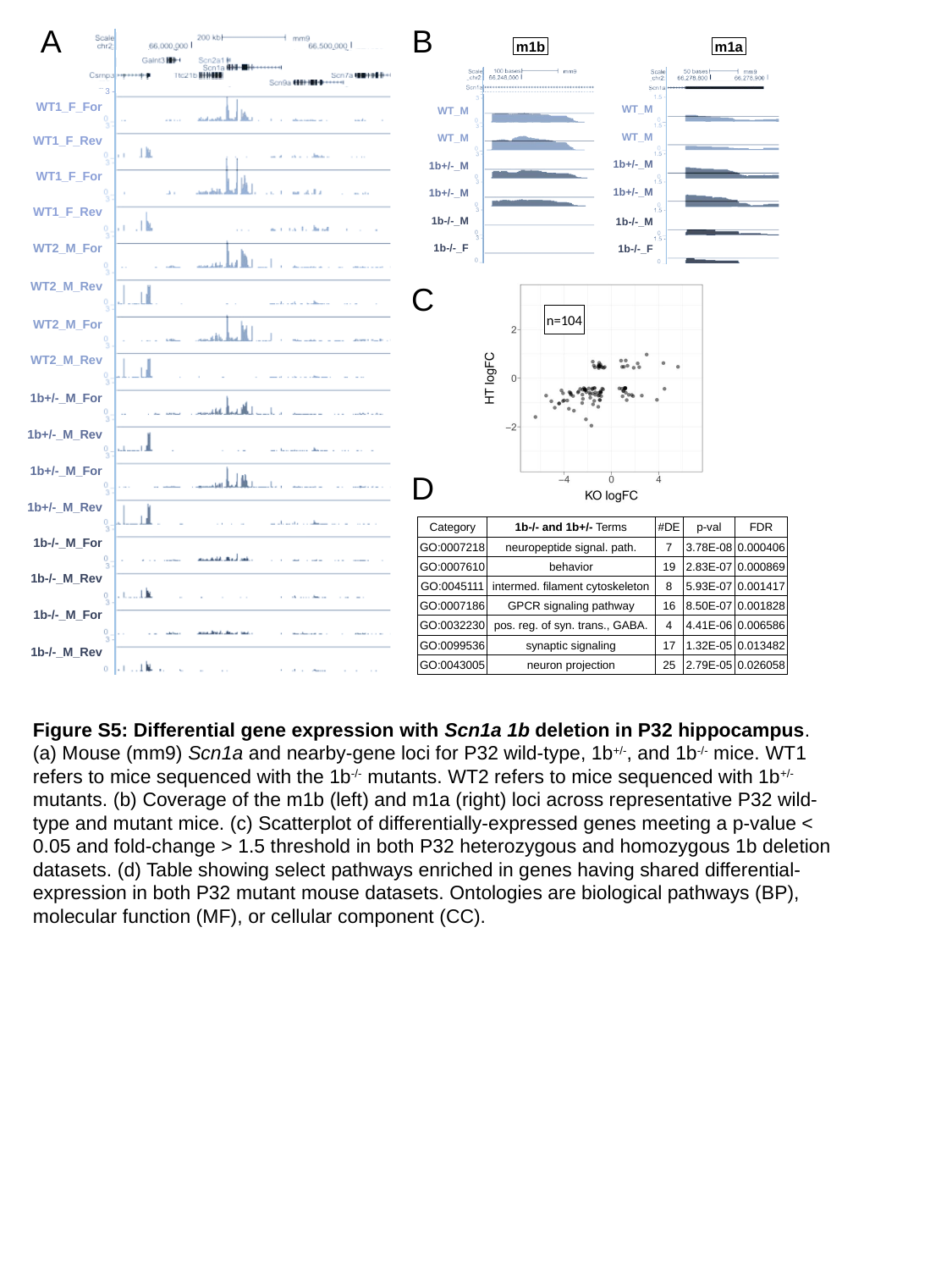

A
B
3 -
WT1_F_For
WT1_F_Rev
WT1_F_For
WT1_F_Rev
WT2_M_For
WT2_M_Rev
WT2_M_For
WT2_M_Rev
1b+/-_M_For
1b+/-_M_Rev
1b+/-_M_For
1b+/-_M_Rev
1b-/-_M_For
1b-/-_M_Rev
1b-/-_M_For
1b-/-_M_Rev
m1a
WT_M
WT_M
1b+/-_M
1b+/-_M
1b-/-_M
1b-/-_F
m1b
WT_M
WT_M
1b+/-_M
1b+/-_M
1b-/-_M
1b-/-_F
C
n=104
D
| Category | 1b-/- and 1b+/- Terms | #DE | p-val | FDR |
| --- | --- | --- | --- | --- |
| GO:0007218 | neuropeptide signal. path. | 7 | 3.78E-08 | 0.000406 |
| GO:0007610 | behavior | 19 | 2.83E-07 | 0.000869 |
| GO:0045111 | intermed. filament cytoskeleton | 8 | 5.93E-07 | 0.001417 |
| GO:0007186 | GPCR signaling pathway | 16 | 8.50E-07 | 0.001828 |
| GO:0032230 | pos. reg. of syn. trans., GABA. | 4 | 4.41E-06 | 0.006586 |
| GO:0099536 | synaptic signaling | 17 | 1.32E-05 | 0.013482 |
| GO:0043005 | neuron projection | 25 | 2.79E-05 | 0.026058 |
Figure S5: Differential gene expression with Scn1a 1b deletion in P32 hippocampus. (a) Mouse (mm9) Scn1a and nearby-gene loci for P32 wild-type, 1b+/-, and 1b-/- mice. WT1 refers to mice sequenced with the 1b-/- mutants. WT2 refers to mice sequenced with 1b+/- mutants. (b) Coverage of the m1b (left) and m1a (right) loci across representative P32 wild-type and mutant mice. (c) Scatterplot of differentially-expressed genes meeting a p-value < 0.05 and fold-change > 1.5 threshold in both P32 heterozygous and homozygous 1b deletion datasets. (d) Table showing select pathways enriched in genes having shared differential-expression in both P32 mutant mouse datasets. Ontologies are biological pathways (BP), molecular function (MF), or cellular component (CC).
