# Supplementary material for: Deletion of a non-canonical regulatory sequence causes loss of Scn1a expression and epileptic phenotypes in mice
Source: Genome Med. 2021 Apr 26;13:69. doi: 10.1186/s13073-021-00884-0 (PMC8080386; doi:10.1186/s13073-021-00884-0)
Supplement: Supplementary file 2 — Additional file 2:. Table S1. Expected Mendelian ratios and observed number of pups born. [file 13073_2021_884_MOESM2_ESM.docx]

**Table S1: Expected Mendelian ratios and observed number of pups born**
The genotypes were born at expected Mendelian ratios, 13 1b^+/-^ x 1b^+/-^ litters dropped from 3 generations of pairings combined. 14 litters were cannibalized.

| Genotypes | Expected | Observed |
| --- | --- | --- |
| 1b^+/-^ | 47 | 52 |
| 1b^-/-^ | 23 | 23 |
| WT | 23 | 19 |
