# Supplementary material for: Deletion of a non-canonical regulatory sequence causes loss of Scn1a expression and epileptic phenotypes in mice
Source: Genome Med. 2021 Apr 26;13:69. doi: 10.1186/s13073-021-00884-0 (PMC8080386; doi:10.1186/s13073-021-00884-0)
Supplement: Supplementary file 3 — Additional file 3:. Table S2. Summary of measures of general health and development in wildtype, heterozygous and homozygous 1b deletion mice. [file 13073_2021_884_MOESM3_ESM.docx]

**Table S2: Summary of measures of general health and development in wildtype and heterozygous 1b deletion mice**

| **Domain** | **Assay** | **Sample size** | **Statistical test** | **Statistic** | **p-value** | **Significant (p<0.05)?** | **Sidak’s multiple comparisons**  **(WT vs 1b^+/-^** **)** | **p-value** |
| --- | --- | --- | --- | --- | --- | --- | --- | --- |
| Physical | Weight | WT N = 24  1b^+/-^ N = 28 | Two Way Repeated Measures ANOVA | F (1, 50) = 0.1063 | p = 0.7457 | No | PND 2 | p > 0.9999 |
|  |  |  |  |  |  |  | PND 4 | p = 0.9998 |
|  |  |  |  |  |  |  | PND 6 | p = 0.9928 |
|  |  |  |  |  |  |  | PND 8 | p = 0.9956 |
|  |  |  |  |  |  |  | PND 10 | p = 0.9518 |
|  |  |  |  |  |  |  | PND 12 | p = 0.9856 |
|  | Total length | WT N = 24  1b^+/-^ N = 28 | Two Way Repeated Measures ANOVA | F (1, 50) = 0.1158 | p = 0.7350 | No | PND 2 | p > 0.9999 |
|  |  |  |  |  |  |  | PND 4 | p = 0.9995 |
|  |  |  |  |  |  |  | PND 6 | p = 0.9998 |
|  |  |  |  |  |  |  | PND 8 | p > 0.9999 |
|  |  |  |  |  |  |  | PND 10 | p > 0.9999 |
|  |  |  |  |  |  |  | PND 12 | p = 0.0931 |
|  | Body length | WT N = 24  1b^+/-^ N = 28 | Two Way Repeated Measures ANOVA | F (1, 50) = 0.01835 | p = 0.8928 | No | PND 2 | p > 0.9999 |
|  |  |  |  |  |  |  | PND 4 | p > 0.9999 |
|  |  |  |  |  |  |  | PND 6 | p = 0.8910 |
|  |  |  |  |  |  |  | PND 8 | p > 0.9999 |
|  |  |  |  |  |  |  | PND 10 | p = 0.9991 |
|  |  |  |  |  |  |  | PND 12 | p = 0.5872 |
|  | Head width | WT N = 24  1b^+/-^ N = 28 | Two Way Repeated Measures ANOVA | F (1, 50) = 0.3327 | p = 0.5667 | No | PND 2 | p = 0.9987 |
|  |  |  |  |  |  |  | PND 4 | p = 0.9953 |
|  |  |  |  |  |  |  | PND 6 | p = 0.9790 |
|  |  |  |  |  |  |  | PND 8 | p = 0.8293 |
|  |  |  |  |  |  |  | PND 10 | p > 0.9999 |
|  |  |  |  |  |  |  | PND 12 | p = 0.9974 |
| Reflex | Negative geotaxis | WT N = 24  1b^+/-^ N = 28 | Two Way Repeated Measures ANOVA | F (1, 50) = 0.04947 | p = 0.8249 | No | PND 2 | p = 0.9921 |
|  |  |  |  |  |  |  | PND 4 | p = 0.3794 |
|  |  |  |  |  |  |  | PND 6 | p = 0.9992 |
|  |  |  |  |  |  |  | PND 8 | p = 0.9994 |
|  |  |  |  |  |  |  | PND 10 | p = 0.9759 |
|  |  |  |  |  |  |  | PND 12 | p = 0.9982 |
|  | Righting reflex | WT N = 24  1b^+/-^ N = 28 | Two Way Repeated Measures ANOVA | F (1, 50) = 0.2493 | p = 0.6198 | No | PND 2 | p = 0.9981 |
|  |  |  |  |  |  |  | PND 4 | p = 0.9791 |
|  |  |  |  |  |  |  | PND 6 | p > 0.9999 |
|  |  |  |  |  |  |  | PND 8 | p = 0.9981 |
|  |  |  |  |  |  |  | PND 10 | p > 0.9999 |
|  |  |  |  |  |  |  | PND 12 | p = 0.9756 |
|  | Circle traverse | WT N = 24  1b^+/-^ N = 28 | Two Way Repeated Measures ANOVA | F (1, 50) = 2.323 | p = 0.1338 | No | PND 2 | p > 0.9999 |
|  |  |  |  |  |  |  | PND 4 | p = 0.9995 |
|  |  |  |  |  |  |  | PND 6 | p = 0.1302 |
|  |  |  |  |  |  |  | PND 8 | p = 0.8712 |
|  |  |  |  |  |  |  | PND 10 | p = 0.9977 |
|  |  |  |  |  |  |  | PND 12 | p = 0.7909 |
|  | Cliff aversion | WT N = 24  1b^+/-^ N = 28 | Two Way Repeated Measures ANOVA | F (1, 50) = 2.225 | p = 0.1421 | No | PND 2 | p = 0.9368 |
|  |  |  |  |  |  |  | PND 4 | p = 0.9303 |
|  |  |  |  |  |  |  | PND 6 | p = 0.1077 |
|  |  |  |  |  |  |  | PND 8 | p = 0.9995 |
|  |  |  |  |  |  |  | PND 10 | p = 0.9997 |
|  |  |  |  |  |  |  | PND 12 | p > 0.9999 |
| Limb strength | Forelimb hang | WT N = 24  1b^+/-^ N = 28 | Two Way Repeated Measures ANOVA | F (1, 50) = 2.704 | p = 0.1064 | No | PND 2 | p > 0.9999 |
|  |  |  |  |  |  |  | PND 4 | p > 0.9999 |
|  |  |  |  |  |  |  | PND 6 | p = 0.4166 |
|  |  |  |  |  |  |  | PND 8 | p > 0.9999 |
|  |  |  |  |  |  |  | PND 10 | p = 0.0158 |
|  |  |  |  |  |  |  | PND 12 | p = 0.9990 |
|  | Hindlimb hang | WT N = 24  1b^+/-^ N = 28 | Two Way Repeated Measures ANOVA | F (1, 50) = 1.946 | p = 0.1692 | No | PND 2 | p = 0.8967 |
|  |  |  |  |  |  |  | PND 4 | p = 0.7164 |
|  |  |  |  |  |  |  | PND 6 | p = 0.9996 |
|  |  |  |  |  |  |  | PND 8 | p = 0.1313 |
|  |  |  |  |  |  |  | PND 10 | p = 0.9614 |
|  |  |  |  |  |  |  | PND 12 | p > 0.9999 |
