# Supplementary material for: Deletion of a non-canonical regulatory sequence causes loss of Scn1a expression and epileptic phenotypes in mice
Source: Genome Med. 2021 Apr 26;13:69. doi: 10.1186/s13073-021-00884-0 (PMC8080386; doi:10.1186/s13073-021-00884-0)
Supplement: Supplementary file 4 — Additional file 4:. Table S3. Summary of the battery of behavioral tests. [file 13073_2021_884_MOESM4_ESM.docx]

**Table S3: Summary of the battery of behavioral tests**

|  | **Assay** | **Metric** | **Sample size** | **Statistical test** | **Statistic** | **p-value** | **Significant (p<0.05)?** | **Sidak’s multiple comparisons**  **WT vs 1b^+/-^** | **p-value** |
| --- | --- | --- | --- | --- | --- | --- | --- | --- | --- |
| Anxiety-like | Elevated-plus maze | %Open arm time | WT = 26  1b^+/-^ = 30 | Unpaired Two-Tailed  T-Test | T (54) = 0.2605 | p = 0.7955 | No |  |  |
|  |  | Total entries | WT = 26  1b^+/-^ = 30 | Unpaired Two-Tailed  T-Test | T (54) = 0.3618 | p = 0.7189 | No |  |  |
|  | Light-dark | Time in dark chamber | WT = 26  1b^+/-^ = 30 | Unpaired Two-Tailed  T-Test | T (54) = 2.067 | **p = 0.0436** | Yes |  |  |
|  |  | Total transitions | WT = 26  1b^+/-^ = 30 | Unpaired Two-Tailed  T-Test | T (54) = 0.9267 | p = 0.3582 | No |  |  |
| Motor | Beam walking | Latency to cross | WT = 25  1b^+/-^ = 29 | Two Way Repeated Measures ANOVA | F (1, 52) = 0.5912 | p = 0.4454 | No | Rod 1 | p = 0.9999 |
|  |  |  |  |  |  |  |  | Rod 2 | p = 0.7336 |
|  |  |  |  |  |  |  |  | Rod 3 | p = 0.7859 |
|  | Rotarod | Latency to fall | WT = 26  1b^+/-^ = 29 | Two Way Repeated Measures ANOVA | F (1, 53) = 1.125 | p = 0.2937 | No | Day 1 | p = 0.3252 |
|  |  |  |  |  |  |  |  | Day 2 | p = 0.4701 |
|  |  |  |  |  |  |  |  | Day 3 | p = 0.9989 |
| Learning and Memory | Fear conditioning | Freeze time -training | WT = 24  1b^+/-^ = 28 | Two Way Repeated Measures ANOVA | F (1, 50) = 0.008692 | p = 0.9261 | No | Pre-training vs Post-training | WT **p < 0.0001** |
|  |  |  |  |  |  |  |  |  | 1b^+/-^ **p < 0.0001** |
|  |  | Freeze time -context | WT = 24  1b^+/-^ = 28 | Unpaired Two-Tailed  T-Test | T (50) = 0.8902 | p = 0.3776 | No |  |  |
|  |  | Freeze time -cue | WT = 24  1b^+/-^ = 28 | Two Way Repeated Measures ANOVA | F (1, 50) = 0.07299 | p = 0.7881 | No | Pre-cue vs Post-cue | WT **p < 0.0001** |
|  |  |  |  |  |  |  |  |  | 1b^+/-^ **p < 0.0001** |
| Social | 3-chambered social approach | Time in chamber | WT = 25  1b^+/-^ = 29 | Two Way Repeated Measures ANOVA | Genotype F (1, 52) = 2.068 | p = 0.1564 | No | Novel object vs Novel mouse | WT **p < 0.0001** |
|  |  |  |  |  | Chamber F (2, 104) = 262.8 | **p<0.0001** | Yes |  |  |
|  |  |  |  |  |  |  |  |  | 1b^+/-^ **p < 0.0001** |
|  |  | Time sniffing | WT = 25  1b^+/-^ = 29 | Two Way Repeated Measures ANOVA | Genotype F (1, 52) = 0.1736 | p = 0.6786 | No  Yes | Novel object vs Novel mouse | WT **p < 0.0001** |
|  |  |  |  |  | Chamber F (1, 52) = 90.48 | **p<0.0001** |  |  | 1b^+/-^ **p < 0.0001** |
|  |  | Transitions | WT = 25  1b^+/-^ = 29 | Two Way Repeated Measures ANOVA | Genotype F (1, 52) = 2.441 | p = 0.1243 | No | WT | p = 0.2881 |
|  |  |  |  |  | Chamber F (2, 104) = 0.6140 | p = 0.4368 | No | 1b^+/-^ | p = 0.9053 |
|  | Male-female social interaction | Nose to nose sniffing | WT = 12  1b^+/-^ = 15 | Unpaired Two-Tailed  T-Test | T (25) = 0.08607 | p = 0.9321 | No |  |  |
|  |  | Nose to anogenital sniffing | WT = 12  1b^+/-^ = 15 | Unpaired Two-Tailed  T-Test | T (25) = 1.374 | p = 0.1817 | No |  |  |
|  |  | Following | WT = 12  1b^+/-^ = 15 | Unpaired Two-Tailed  T-Test | T (25) = 0.2615 | p = 0.7958 | No |  |  |
|  |  | Exploring | WT = 12  1b^+/-^ = 15 | Unpaired Two-Tailed  T-Test | T (25) = 1.009 | p = 0.3224 | No |  |  |
|  |  | Sum ultrasonic vocalizations | WT = 12  1b^+/-^ = 15 | Unpaired Two-Tailed  T-Test | T (25) = 2.143 | p = 0.0420 | Yes |  |  |
| Repetitive | Self-groom | Time grooming | WT = 26  1b^+/-^ = 30 | Unpaired Two-Tailed  T-Test | T (54) = 0.2345 | p = 0.8155 | No |  |  |
| Sensorymotor reflex | Acoustic startle | Startle amplitude | WT = 26  1b^+/-^ = 27 | Two Way Repeated Measures ANOVA | F (1, 51) = 0.3275 | p = 0.5696 | No |  |  |
|  | Pre-pulse inhibition | Startle amplitude | WT = 26  1b^+/-^ = 27 | Two Way Repeated Measures ANOVA | F (1, 51) = 0.3244 | p = 0.5715 | No |  |  |
