# Supplementary material for: Deletion of a non-canonical regulatory sequence causes loss of Scn1a expression and epileptic phenotypes in mice
Source: Genome Med. 2021 Apr 26;13:69. doi: 10.1186/s13073-021-00884-0 (PMC8080386; doi:10.1186/s13073-021-00884-0)
Supplement: Supplementary file 5 — Additional file 5:. Table S4. Lack of sex difference between wildtype, 1b+/− and 1b−/− mice in behavioral assays. [file 13073_2021_884_MOESM5_ESM.docx]

**Table S4: Lack of sex difference between wildtype, 1b^+/-^ and 1b^-/-^ mice in behavioral assays**

| **Assay** | **Metric** | **Sample size** | **Statistical test** | **Statistic** | **p-value** | **Significant (p<0.05)?** |
| --- | --- | --- | --- | --- | --- | --- |
| Open field | Horizontal activity | WT M = 12  WT F = 14 | Two Way Repeated Measures ANOVA | F (1, 24) = 2.874 | p = 0.1030 | No |
|  |  | 1b^+/-^ M = 15  1b^+/-^ F = 15 | Two Way Repeated Measures ANOVA | F (1,28) = 3.225 | p = 0.0833 | No |
|  |  | 1b^-/-^ M = 6  1b^-/-^ F = 6 | Two Way Repeated Measures ANOVA | F (1, 10) = 0.2820 | p = 0.6070 | No |
|  | Vertical activity | WT M = 12  WT F = 14 | Two Way Repeated Measures ANOVA | F (1, 24) = 1.549 | p = 0.2253 | No |
|  |  | 1b^+/-^ M = 15  1b^+/-^ F = 15 | Two Way Repeated Measures ANOVA | F (1,28) = 0.1632 | p = 0.6893 | No |
|  |  | 1b^-/-^ M = 6  1b^-/-^ F = 6 | Two Way Repeated Measures ANOVA | F (1, 10) = 4.398 | p = 0.0624 | No |
|  | Total activity | WT M = 12  WT F = 14 | Two Way Repeated Measures ANOVA | F (1, 24) = 3.477 | p = 0.0745 | No |
|  |  | 1b^+/-^ M = 15  1b^+/-^ F = 15 | Two Way Repeated Measures ANOVA | F (1,28) = 1.627 | p = 0.2125 | No |
|  |  | 1b^-/-^ M = 6  1b^-/-^ F = 6 | Two Way Repeated Measures ANOVA | F (1, 10) = 7.226 | **p = 0.0228*** | Yes |
| Novel Object Recognition | Novel-Familiar sniff  Novel phase | WT M = 12  WT F = 14 | Unpaired Two-Tailed  T-Test | T (24) = 1.497 | p = 0.1475 | No |
|  |  | 1b^+/-^ M = 15  1b^+/-^ F = 15 | Unpaired Two-Tailed  T-Test | T (28) = 0.3861 | p = 0.7023 | No |
|  |  | 1b^-/-^ M = 6  1b^-/-^ F = 6 | Unpaired Two-Tailed  T-Test | T (10) = 1.764 | p = 0.1081 | No |
| Spontaneous alternation | % Spontaneous alternation | WT M = 12  WT F = 13 | Unpaired Two-Tailed  T-Test | T (23) = 1.176 | p = 0.2516 | No |
|  |  | 1b^+/-^ M = 15  1b^+/-^ F = 14 | Unpaired Two-Tailed  T-Test | T (27) = 0.5110 | p = 0.6135 | No |
|  |  | 1b^-/-^ M = 6  1b^-/-^ F = 5 | Unpaired Two-Tailed  T-Test | T (9) = 0.8541 | p = 0.4152 | No |
